# Development of ACT+: A Novel, Person‐Centred Psychological Intervention Based on Acceptance and Commitment Therapy (ACT) to Improve Quality of Life in Patients Living With and Beyond Cancer

**DOI:** 10.1111/hex.70237

**Published:** 2025-03-26

**Authors:** Elisavet Moschopoulou, Sheila Donovan, Debbie Brewin, Stephanie Taylor, Damien Ridge, Liam Bourke, Imran Khan, Moïse Roche, Trudie Chalder

**Affiliations:** ^1^ Barts and The London School of Medicine and Dentistry Queen Mary University of London London UK; ^2^ Department of Psychological Medicine Kings College London London UK; ^3^ College of Liberal Arts & Sciences University of Westminster London UK; ^4^ School of Health and Social Care Sheffield Hallam University Sheffield UK; ^5^ Division of Psychiatry University College London London UK

**Keywords:** acceptance and commitment therapy, living with and beyond cancer, patient and public involvement, person‐centred, psychological intervention, qualitative

## Abstract

**Introduction:**

The need to improve the quality of life (QoL) and well‐being of people living with and beyond cancer is well recognised. SURECAN (SUrvivors' Rehabilitation Evaluation after CANcer) is a multiphase study developing and evaluating a psychological intervention based on acceptance and commitment therapy (ACT) for people who have completed hospital‐based treatment for cancer but have low QoL. We describe the process by which we iteratively developed and refined ACT+, an intervention in which ACT is integrated with options to support exercise and work.

**Methods:**

ACT+ development was guided by the theory of ACT while Normalisation Process Theory (NPT) was used as a sensitising tool at all stages. Evidence from several strands of research comprising a qualitative meta‐synthesis, a qualitative study with stakeholders and pre‐pilot testing was brought together to refine ACT+. Insights from patient and public involvement (PPI) consultations supported the development and refinement of ACT+ resources throughout.

**Results:**

The qualitative study with stakeholders shed light on the ‘real world’ contexts in which the ACT+ intervention would be offered and accessed, as well as the appeal of ACT+ as a therapy for people living with and beyond cancer. People who had treatment for cancer (*n* = 31) and healthcare professionals (*n* = 16) provided overall support for the intervention. Subsequent pre‐pilot testing of the intervention and qualitative work with cancer patients (*n* = 6) and therapists (*n* = 7) led to further refinements. Evidence collected from all strands of research and PPI was integrated in an iterative way to produce an intervention that was acceptable to all.

**Conclusion:**

We adopted an iterative and evidence‐based approach to the development of the ACT+ intervention, which was acceptable to patients and healthcare professionals. Future work will examine the effectiveness of ACT+.

**Patient and Public Engagement:**

This intervention was developed and refined with substantial involvement from the study's patient and public involvement group and others accessed via community/patient groups to discuss and gain feedback on patient‐facing materials. For example, the ACT+ participant handbook underwent four consultation rounds with patient representatives, including a consultation specifically focused on its cultural acceptability. Therefore, emerging insights from PPI were also used to optimise ACT+ components and delivery. Their input was integrated throughout all three strands of the research.

## 1 Background

There are currently 3 million people living with and beyond cancer in the United Kingdom [1, 2]. With advances in cancer diagnosis and treatment, survival rates are steadily increasing [1, 3], with 50% of patients now surviving cancer by 10 or more years [1, 4]. About a third report a diminished quality of life (QoL) [5, 6, 7] due to the physical and psychological effects of the disease and its treatment. Furthermore, rates of psychological distress are higher in cancer survivors compared to the general population, with estimates ranging between 14% and 24% for clinical depression and 10% for clinical anxiety, depending on factors such as the type and stage of cancer, as well as the time since treatment completion [Fardell et al. 2023; Mitchell et al. 2013; Niedzwiedz et al. 2019]. A national survey assessing the QoL of adult cancer survivors in England reported key issues such as fear of cancer recurrence (57%), higher levels of fatigue (43%), body image concerns (31%) and lack of exercise (30%) [5]. Poor QoL is also associated with unemployment in those of working age [8], with up to a third losing their employment after cancer [5]. Importantly, previous research emphasises the significance of work in terms of providing structure and purpose in daily life and enabling patients to gain a sense of normality after their diagnosis and treatment [Eva et al. 2012; Rasmussen et al. 2008; Kennedy et al. 2007].

As the number of patients living with and beyond cancer increases, the need for evidence‐based interventions to support QoL becomes imperative. Several policy documents have emphasised the importance of cancer survivorship and improving QoL outcomes, two areas that are also recognised as important targets in the NHS Long Term Plan for Cancer (https://www.england.nhs.uk/cancer/strategy). The 2015 NHS Independent Cancer Taskforce report recommended ‘making quality of life a priority’ and included in their goals that every person with cancer should have access to holistic support through a ‘recovery package’ of aftercare with ‘stratified pathways of follow‐up care’ [7]. The report also recommended that return to work is fully integrated into care planning and support [7]. Similarly, the National Cancer Survivorship Initiative (NCSI) has included in its UK top 10 living‐with‐and‐beyond‐cancer research priorities the need to identify the most effective ways of supporting the psychological well‐being of all people affected by cancer, their carers and families [9].

There is wide variation in NHS ‘aftercare’ [6, 10], with no universal or consistent provision of specific interventions or therapies. The results of a national survey of post‐cancer treatment follow‐up care in the UK showed that the most common interventions offered were dietary (72%) and exercise (65%) advice, as well as a one‐off ‘end of care’ intervention (62%), and counselling (61%) [10]. Evidence‐based approaches like Cognitive Behavioural Therapy (CBT) (16%), mindfulness (21%) and return‐to‐work support (20%) were among the least frequently offered, often due to resource constraints, inconsistent commissioning and limited training for healthcare professionals (HCPs) [10, 11]. Our previous systematic review examined non‐pharmacological interventions aimed at improving cancer survivors' QoL and suggested that only CBT, or exercise‐based interventions, were moderately effective [11]. We found limited evidence for acceptance and commitment therapy (ACT) in cancer [12], although preliminary evidence from other chronic diseases and long‐term conditions (e.g., epilepsy, paediatric illness, multiple sclerosis and diabetes) suggested that ACT may be beneficial in improving QoL, reducing distress, enhancing symptom control and supporting disease self‐management [13].

ACT is grounded in Relational Frame Theory (RFT), a modern behavioural theory of human language, and is an empirically based psychological intervention that aims to enhance ‘psychological flexibility’—the ability to adapt to demands, shift perspectives and balance competing desires and needs [14]. Originally developed to address psychological disorders such as anxiety and depression, ACT does not focus on reducing unwanted symptoms and thoughts. Instead, it encourages individuals to accept difficult experiences, clarify personal values and commit to values‐driven actions [14, 15]. ACT lends itself to addressing the concerns of patients living with and beyond cancer, in helping patients to accept what cannot be changed (e.g., a possible cancer recurrence) while committing to pursuing realistic and meaningful change in line with their personal values. It is personalised and flexible enough to be helpful for patients with a range of concerns and has the potential to transcend clinical and/or socio‐cultural differences.

To address the lack of robust evidence in cancer support, and improve patients' QoL, we developed a theory‐driven intervention based on ACT. Given the well‐documented benefits of exercise and the positive impact of meaningful occupation (i.e., paid work or unpaid activities) for people living with and beyond cancer, we adapted ACT to incorporate options for supporting these areas relevant to individuals' values and goals. Our intervention, called ACT+, was designed for people with a range of cancers (i.e., head and neck, breast, lower gastro‐intestinal, urological and haematological) who had completed hospital‐based treatment for cancer with curative intent but continued to experience poor QoL.

We describe the process by which the intervention was co‐developed and refined. Several strands of research were carried out alongside one another and fed into each other in an iterative process. This study drew on the theory of ACT and stakeholder input guided by Normalisation Process Theory (NPT) [16]. The overarching aims were to develop an intervention that was:

1. intended to improve QoL.
2. suitable for different cancer groups.
3. integrated with options to support physical activity and meaningful occupation.
4. (including the return to paid employment), where this was compatible with individuals' personal values and goals.
5. culturally acceptable to different groups in the United Kingdom.

## 2 Methods

We present the development of ACT+ in three strands of work. This paper provides an overview of all strands of the research conducted, with a detailed report on the findings from Strand 3.

The NPT framework can be used to guide the development of interventions to facilitate implementation in real‐world settings [16]. We applied NPT constructs—specifically context, coherence, cognitive participation, collective action and reflective monitoring—as a ‘sensitising framework’ throughout the development, refinement and piloting of ACT+. For example, the ‘context’ construct guided our exploration of the practical considerations involved in delivering ACT+, ensuring its compatibility with existing practices. ‘Coherence’ helped us think through issues of communication, ensuring that the intervention and trial processes were clearly explained and understood by both patients and HCPs. ‘Cognitive participation’ informed our efforts to engage key stakeholders and encourage their involvement with ACT+, while ‘collective action’ prompted us to consider how ACT+ could be integrated into routine NHS workflows. Finally, ‘reflexive monitoring’ ensured that stakeholder feedback was used to refine both the intervention content and trial procedures. Table 1 outlines the guiding questions we applied using these constructs. Additionally, NPT informed the design of the topic guides used in interviews and focus groups with key stakeholders during the ACT+ development phase (see Supplementary Information File 1). This approach aligns with the latest MRC guidance for developing complex interventions [17], which focuses on six elements (i.e., context; developing, refining and testing programme theory; engaging stakeholders; identifying key uncertainties; refining intervention and economic considerations).

**Table 1:** Normalisation process theory (NPT) constructs and how they were used in the intervention development and refinement process.

NPT component	Sample ACT+ development questions	Sample ACT+ refinement questions	Sample ACT+ evaluation/testing questions
Context	Can the intervention contexts be described? Which staff are affected? What are the immediate and long‐term concerns of staff?	What are the practical issues in delivering ACT+?	How does the ACT+ trial fit with systems already in place?
Coherence	Can the intervention be easily described?	To what extent do users share in the ACT+ sense of purpose?	Can the trial be understood? To what extent do staff share in the ACT+ trial vision?
Cognitive participation	Do user groups including patients of different ethnicities/genders/cancers see the point of the intervention?	How are users affected by initial delivery and can their experience be improved? Can training be improved to better engage staff and users?	To what extent do staff understand the rationale for the trial?
Collective action	How compatible is ACT+ with existing practices? How might ACT+ affect health consultations?	How might ACT+ promote and/or impede usual NHS work?	How can ACT+ fit into organisations post‐trial?
Reflexive monitoring	How can perceived advantages of ACT+ be maximised? How can users/staff best contribute feedback to refine ACT+?	How do we refine ACT+ content and delivery of stakeholder experience?	How do we refine the ACT+ trial practicalities of stakeholder experience?

### 2.1 Strand 1—Initial Development of a Theory Based‐Intervention

We believed that the theory associated with ACT, that is, psychological flexibility, would be the foundation on which to build the ACT+ intervention. The integration of exercise and work/meaningful occupation support were also deemed to be important, but consideration was given as to how they might be integrated into ACT+ without losing the integrity of ACT. We discussed these ideas with our patient representatives who liked the theory of ACT and could see the potential benefit to people with cancer who had been treated successfully. Theory‐based logic modelling was used to map the existing evidence and key intervention mechanisms onto suggested behaviour change processes and intervention outcomes. See Supplementary Information File 2 for a summarised logic model for ACT+.

Preliminary versions of a study‐specific therapist manual and a handbook for participants were drafted based on the theory of ACT with a view to piloting ACT+. These resources were then shared with patient representatives for feedback. Consideration at this stage was given to therapists' needs, as well as to issues of fidelity. Therefore, an initial training programme was developed for therapists, based on the team's extensive experience in ACT and training in complex interventions. In terms of who would be best placed to deliver ACT+ in England, the study team agreed that therapists could be situated in NHS Talking Therapies (formerly known as Improving Access to Psychological Therapies [IAPT]), specialist services or cancer charities. Our decision was guided by the fact that NHS Talking Therapies, a National Health Service (England) initiative, had recently widened its scope to provide therapy to people with long‐term physical health conditions, including cancer, with emotional distress. Taking part in the study would mutually benefit the SURECAN study and the services involved by having some of their therapists trained in ACT—a model they could use with other patients in their service. Traditionally, they offer evidence‐based therapies to support adults experiencing depression and anxiety. Therapists would include clinical psychologists, high‐intensity therapists (from NHS Talking Therapies) and counsellors. The sessions would be delivered at their respective practices face‐to‐face, by phone or via online video calling, for example, Skype and so forth, to suit individual needs. We initially agreed that ACT+ could take the form of 6 sessions at weekly or fortnightly intervals. To improve adherence to the intervention, therapists would be offered monthly group supervision, as well as individual advice and support if required.

### 2.2 Strand 2—Refinement of the Intervention and Intervention Materials

a. **Meta‐ethnography study**: Understanding how to shape ACT+ to be more acceptable in a culturally diverse society was considered essential from the outset. Therefore, in parallel with the preparation of preliminary intervention materials, we conducted a qualitative meta‐ethnographic synthesis based on the methods of Noblit and Hare [18], to investigate potential social and cultural factors for cancer‐related psychological interventions. Preliminary findings from the meta‐ethnography [19] were fed back to the core intervention development team (E.M., D.B. and T.C.), who identified key elements that would help improve the content and delivery of ACT+.
b. **Qualitative study with key stakeholders:** Having developed a preliminary outline for the intervention, we conducted exploratory qualitative interviews and focus groups with cancer patients and HCPs to gather their views on the proposed intervention.
c. **ACT+ training:** In preparation for a pre‐pilot test run of the proposed intervention, we recruited and trained therapists from different services to deliver ACT+ sessions to cancer patients. ACT+ training was delivered via interactive workshops conducted over 2–3 days by two experienced cognitive behavioural therapists with extensive clinical and training experience in ACT. Three workshops were delivered to a total of 29 therapists (Cognitive Behaviour Therapists, Counselling Psychologists and Clinical Psychologists) from three clinical settings in London and Sheffield. Feedback was collected from all trained therapists to explore their experience and modify the programme accordingly. Semi‐structured interviews were conducted with a purposive sample of therapists (*n* = 12) to explore their views about the training more deeply. The aim was to investigate how effective training was at improving therapists' knowledge and confidence to deliver the manualised ACT+ intervention and to understand how training might be optimised. Details regarding the development of the ACT+ training programme and its subsequent evaluation have been published separately [20].

Throughout the intervention refinement process, the core intervention development team (E.M., D.B. and T.C.) reviewed emerging insights from different sources of evidence, that is, meta‐ethnography and qualitative work, as well as insights from the wider management team discussions and feedback from patient representatives to optimise key intervention features (e.g., content, materials, training and delivery) to improve its appeal and acceptability. A record of key intervention and training considerations and decisions was kept (see Supplementary Information File 3).

#### 2.2.1 Qualitative Study Methodology

We conducted interviews (individual and group) with two groups of SURECAN study stakeholders to elicit responses to the ACT+ intervention: people who reflected the target group for the ACT+ intervention (Group 1) and staff working in NHS services from where participants for the trial would be recruited, or the ACT+ intervention would be delivered (Group 2). A purposive sampling strategy, using maximum variation, was adopted to obtain views from patients of different ages, genders, cultural groups and cancer treatment groups. Similarly, purposive sampling was used for interviews with HCPs to achieve variation in relation to cancer clinics and professions.

Group 1 comprised patients who were within 12 months of completing treatment with curative intent for one of the five cancers targeted in SURECAN. Eleven interviews and four focus groups, all face‐to‐face, were conducted with a total of 31 patients and 2 family carers (each of whom had accompanied a patient participant and consented to be interviewed with them). Patients were recruited via cancer clinics at two of the study research sites (*n* = 29) and a cancer charity affiliated with one of the sites (*n* = 2). Focus groups and interviews took place at the two research sites (*n* = 5), the affiliated cancer charity (*n* = 8) and the study team's base in the university (*n* = 2). Once written consent had been obtained, participants were invited to complete an anonymous form (i.e., with no patient identifier) regarding cancer type and demographics (gender, age band and ethnicity). These data were collated and reviewed to enable us to monitor the diversity of the sample as recruitment progressed. Each focus group/interview started with a PowerPoint presentation situating ACT+ in the context of the SURECAN study and describing ACT+ and the rationale for choosing this approach to aftercare. After this, participants were asked about their understanding of ACT+ and how useful it might be to them and others in a similar situation, potential challenges for using ACT+ and the integrated options on exercise and work/meaningful occupation. In all focus groups and in most interviews, participants were shown one or two of the ACT+ resources (e.g., values cards) and asked for their views. At the end of the focus group/interview, participants were offered a £30 high street shopping voucher.

Participants in Group 2 were HCPs working in cancer clinics (*n* = 11) and psychologists/therapists working in psychological services or psycho‐oncology services (*n* = 5) at study research sites. Eight individual interviews, one two‐person and two three‐person interviews, all face‐to‐face, were conducted with a total of 16 staff. Participants in the two‐ and three‐person interviews were colleagues working in the same cancer clinic or psychological service. Interviews were conducted at the participants' workplace (*n* = 10) and the university (*n* = 1). Interviews started with a PowerPoint presentation about ACT+ and the SURECAN study. Participants were asked for their views on the study and the extent to which ACT+ makes sense, the integrated options and how useful ACT+ might be for patients.

Interviews were digitally audio‐recorded and professionally transcribed. Transcripts were checked for accuracy, anonymised and uploaded to NVivo, where they were coded. A coding framework was developed in an iterative process. An initial draft was developed by S.D. after reading several transcripts multiple times and discussing with the team. A priori codes were included, and new codes were added as the analysis progressed. Summaries of transcripts (of interviews with Group 2 participants) were produced by the team to identify issues/important insights relevant to intervention development and/or trial conduct. Summaries of a sample of transcripts (of interviews with Group 1 participants) were discussed with the team. A reflexive thematic analysis was conducted by S.D., supported by regular discussion with a co‐researcher (E.M.), who had conducted some interviews and co‐facilitated some focus groups. The preliminary and final themes were discussed with the wider team. The findings were drafted by S.D. and reviewed by the qualitative lead (D.R.). Ethical approval was obtained by Cornwall & Plymouth Research Ethics Committee (reference number 18/SW/0196).

### 2.3 Strand 3—Intervention Pre‐Pilot Testing

Following the development and refinement of intervention materials (i.e., research strands 1 and 2), we conducted a small test run of the intervention. The aim was to improve training for therapists and the experience of patients, optimise features of the intervention and ensure the trial research protocol was agreeable to key stakeholders before formal pilot testing.

Eight patients who were within 24 months of completing hospital‐based treatment for cancer with curative intent and had low QoL (defined as a score of 78 or less on the Functional Assessment of Cancer Therapy: General scale) were recruited via clinics at two of the study research sites. Eight trained therapists delivered ACT+ sessions to one patient participant each. Therapy sessions were audio‐recorded.

Qualitative study: Post‐ACT+ delivery, individual semi‐structured interviews were conducted with therapists (*n* = 7/8) and patients (*n* = 6/8) to explore experiences of delivering and receiving ACT+ sessions. Topic guides were developed to cover issues such as the acceptability and usefulness of the intervention, potential improvements, and the feasibility of delivering ACT+. Interviews were audio‐recorded and transcribed verbatim. Anonymised transcripts were uploaded onto NVivo and analysed thematically by S.D. and E.M. Transcript summaries were produced and discussed with the team. Findings were drafted by S.D. and E.M. and reviewed by the study team and the qualitative lead (D.R.).

### 2.4 PPI

Two people with experience of cancer were part of the research team throughout. The value of incorporating patient and public involvement (PPI) feedback alongside qualitative research at all stages of intervention development is well documented and has been proposed to increase the usefulness and acceptability of interventions [21]. Substantial input from the study's PPI group, as well as other patient representatives accessed via community networks, was used to shape and optimise the ACT+ intervention and patient‐facing materials, including the participant handbook.

The SURECAN PPI group consisted of three women and two men, representing a range of cancer types, including breast, prostate and colorectal cancers. The group also included a carer representative and a representative from a minority ethnic background. While most members of the PPI group were of White ethnic background, one participant was Afro‐Caribbean and another was South Asian. To ensure diverse involvement in the study, we also advertised involvement opportunities through existing patient networks, specifically inviting individuals from minority ethnic backgrounds to contribute. This approach successfully engaged people from a broad range of cultural and ethnic backgrounds. For example, the ACT+ participant handbook underwent four consultation rounds with patient representatives, including a consultation with seven patient representatives from a minority ethnic background dedicated to its cultural acceptability. Emerging insights from PPI contributors were used throughout all three strands of the research to inform and optimise the content and delivery of ACT+.

## 3 Results

### 3.1 Strand 1—Initial Development of a Theory Based‐Intervention

Having agreed on key intervention features such as the mode of delivery and the number of sessions, intervention resources were iteratively developed. We wanted them to be used flexibly and to be as personalised as possible. Resources were split into two levels: (i) patient‐facing (i.e., participant handbook) and (ii) therapist‐facing (i.e., therapist manual).

The participant handbook was organised into six chapters covering a range of topics (see Table 2). Additional information on building up exercise and work/meaningful occupation was specifically added in line with the ACT+ approach. Each chapter was developed to include some reading and tasks (e.g., reflective and/or mindfulness exercises) for participants to complete as they read through or between sessions. The aim was to supplement the content of the ACT+ therapy sessions and promote engagement in therapy. At the same time, we aim to use this resource flexibly, depending on individual needs. Therefore, the format we chose was that at the end of each session, the therapist, together with the participant, would decide which chapter to focus on for the next session. Participants did not need to go through all the chapters in the handbook.

**Table 2:** Summary of ACT+ intervention patient‐facing content.

ACT+ process of therapy and sessions	Participant handbook chapters	Aims of sessions
Stage 1: Assessment, engagement and planning of treatment (Sessions 1 and 2)	Chapter 1—Becoming Open: Introduction to ACT and how it can help. Background reading: Introduction to Acceptance and Commitment Therapy (ACT) Walking in the rain—Doing things in the presence of difficulties. Self‐reflection exercise: Open, Aware, Active—The essence of ACT Mindfulness Exercise: Open and Observing—Tuning In More about ACT: Tug of War metaphor Suggested tasks between sessions	To assess the nature of the problems, establish a sound therapeutic alliance and describe the ACT approach. Also, to review between sessions tasks (all sessions), reinforce the message of how ACT works and introduce mindfulness.
Stage 2: Active treatment (Sessions 3–6)	Chapter 2—Exploring Values and Becoming Aware: What is important to me?	To introduce values and goals and what gets in the way, as well as to try out some worksheets such as the ‘what works plan’ and values cards.
	Chapter 3—Becoming Active: Linking values to goals and taking action.	To re‐visit setting values‐based goals in relation to all life areas including exercise and work/occupation if these are identified as important by the participant.
	Chapter 4—Making it personal: skills to overcome challenges and stuck loops.	To review progress to date, explore what keeps the participant stuck (if indeed they are), to reflect on and use a range of ACT‐consistent techniques to increase acceptance.
Stage 3: Preparation for discharge (Sessions 7 and 8)	Chapter 5—Putting it all together: Noticing what helps and maintaining momentum.	To introduce the idea of the cycle of change and what helps keep motivation going and encourage good choices which fit with values. To reflect on what has helped and discuss how to deal with setbacks
	Chapter 6—Looking to the future and taking things forward.	To review gains made in therapy, consider how to maintain flexibility and strengthen self‐care looking to the future. To consider what flexibility and self‐care mean and which parts of the programme helped the participant become flexible.

Similarly, the ACT+ therapist manual was developed to provide information on the experience of cancer and some of the difficulties people faced, an overview of the theory of ACT, including strategies used to promote psychological flexibility, as well as guidance on what might be addressed during therapy. Our aim was for the manual to be a flexible guide to help therapists adopt a broad ACT‐consistent approach. For example, although ACT+ sessions follow a structure (see Table 2 for an overview of the ACT+ process of therapy and session aims), therapists were encouraged to use a formulation‐based approach and to tailor the proposed ACT+ session plans according to participant needs. Study‐specific therapy aids and materials (e.g., metaphors and mindfulness exercises) were provided, although their use was not intended to be prescriptive. As we anticipated that therapists might need some additional guidance to support the possible inclusion of exercise/work‐related goals, the material was developed and included specifically to cover these topics.

### 3.2 Strand 2—Refinement of the Intervention and Intervention Materials

a. **Meta‐ethnography study:** In a series of analytical sessions/meetings involving the core meta‐ethnography team (led by D.R.), as well as the core intervention development team (led by T.C.) and the wider study team, preliminary recurring themes identified in the literature were presented, discussed and debated. Several intervention considerations were discussed and agreed upon. For example, emphasis was placed on ensuring that the language that was used in the delivery of the intervention, that is, written materials and in‐session delivery, was acceptable to people from ethnically and culturally diverse communities. Drawing on the results of the meta‐synthesis, an additional session was included in the ACT+ therapist training programme to highlight important topics in relation to adapting therapy for racially minoritised patients, such as the importance of empathy and providing unconditional positive regard (no judgement, respect, sensitivity towards people's religious beliefs and take people at face value), the role of stigma, being curious about alternative health belief systems and being aware of potential language and communication challenges. We emphasised the person‐centred approach allowing participants to be seen, as a whole person. This is at the core of ACT, but we wanted to emphasise how we are positioned in relation to others, highlighting the need for curiosity about cultural differences and putting complex issues around race on the table for discussion. We, therefore, added 2 additional sessions to the original 6 and modified our training.
b. **Qualitative study with stakeholders (patients and HCPs)**: Patients from a wide range of cancers participated (breast *n* = 7, colorectal *n* = 6, haem‐oncology *n* = 5, head and neck *n* = 5, prostate *n* = 8). Twenty‐one were male. The majority were white, aged 35 onwards (see Table 3 for details). HCPs comprised 8 clinical nurse specialists, 2 consultants, 1 allied HCP and 5 clinical psychologists/therapists.
  From the analysis, three themes were identified. The first, ‘post‐treatment cancer care and ACT+’, highlights the cancer care pathway and provides the context for locating ACT+ in the trajectory of care. In the second theme, ‘candidacy for ACT+’, the focus is on the individual for whom ACT+ could be beneficial. The third theme, ‘ACT+ as aftercare’, with the sub‐theme ‘ACT+ integrated options’, sheds light on the appeal of ACT+ as a therapy for people living with and beyond cancer. These results are described in detail in Table 4.

**Table 3:** Demographic and cancer characteristics of patient participants (Strand 2).

Cancer type/gender			
	Total	Female	Male
Breast	7	7	
Colorectal	6	2	4
Haem‐oncology	5		5
Head and neck	5	1	4
Prostate	8		8
Total	31	10	21

Cancer type/age/gender													
Cancer type	Total	Age band (years)											
		15–24		25–34		35–44		45–54		55–64		65+	
		F	M	F	M	F	M	F	M	F	M	F	M
Breast	7					2				3		2	
Colorectal	6							1	1		1	1	2
Haem‐oncology	5						2		2		1		
Head and neck	5										1	1	3
Prostate	8										3		5
Total	31	—		—		4		4		9		14	

Cancer type/ethnic group						
Cancer type	Total	White	Mixed/Multiple ethnic groups	Asian/Asian British	Black/African/Caribbean/Black British	Other ethnic group
Breast	7	5 (British)	1 (Other Mixed/Multiple background)	1 (Other Asian background)		
Colorectal	6	6 (British)				
Haem‐oncology	5	3 (British) 2 (Other White background)				
Head and neck	5	5 (British)				
Prostate	8	5 (British) 1 (Irish)		1 (Pakistani) 1 (Other Asian background)		
Total	31	27	1	3	—	—

**Table 4:** Qualitative study results which informed intervention development: themes and quotes.

Theme	Theme description	Illustrative quotations
Post‐treatment cancer care and ACT+	Patients and healthcare professionals highlighted that the end of treatment signalled important changes in cancer care, where support for patients could be lacking. Patients wanted to know how they could access ACT+ therapy and the circumstances in which it might be offered. Both patients and HCPs saw benefits in ACT+ being incorporated into the cancer care pathway.	*But cancer survivors unfortunately I think lack the support afterwards. They have intense input for a number of weeks…And then after treatment it doesn't happen very much. (HCP.Int 1)*
		*Because you feel the rug [being pulled] when the treatment ends.…Because you've been so dependent. In my treatment I had chemo weekly, and I could hardly walk by the end of it. So, the hospital is my world, I came every Monday and had more. And it's very traumatic. I mean you want it to stop, and then it stops, and you feel abandoned. So, it's very strange. (PAT.Int 4)*
		*So, in my situation at what point would that process happen? So, I went today for example to see [Name of consultant] and 50 seconds later you're feeling fine, got a PSA of 0.01, that's great, undetectable, see you in six months' time. I didn't see anybody else. […] So, at what point would somebody pick that up, that maybe I've got a psychological issue, I've got a problem, or I feel anything other than…what I've actually said? (PAT.FG4, R4)*
		*I think it would be a good thing to be able to refer people onto it at the end of the treatment, rather than just saying if you need us, ring us. (HCP.Int 5)*
Candidacy for ACT+	Candidacy for ACT+ was conceptualised in two ways in the interviews: as eligibility for a study intervention and receptivity to the idea of talking therapy. In terms of who would be eligible, some patients and HCPs raised concerns about how quality of life would be assessed. Regarding engaging in talking therapy, participants identified groups of patients who would likely not engage such as those from ethnic groups who ‘don't like to…talk about their feelings or their fears’, or who ‘just want to keep things in the family’, and those preferring to get help from their family or from their religious pastors. The stigma associated with talking therapies was also highlighted by psychologists/HCPs.	*And also, what do you think is well‐being? Is well‐being happiness or is well‐being feeling fit and healthy? There're so many aspects of what well‐being could be. (HCP.Int 10, R1)*
		*I mean I've done questionnaires for various things, health and wellbeing stuff and that type of thing, where it asks you how do you feel today? And that's got…I mean basically I'm a pretty positive person. So generally, that's a fairly high score. But if it caught me on a bad day, it could be… (PAT.Int 5, R2)*
		*If we had spoken two years ago, I would have said oh, it's a load of rubbish and a waste of time. Because I've never been that kind of person. But having been through it I'm a total advocate. I think it's fantastic. But you've got to be receptive to it. (PAT.FG2, R2)*
ACT+ as aftercare	For patients, the appeal of ACT+ was spoken about in terms of being able to look ahead (their onward journey) which had not been a focus of the treatment phase (*FG2, R3*). As an aftercare therapy tailored to individual values and needs, ACT+ was particularly appealing in contrast to the relatively standardised care during cancer treatment (*FG3, R2*). For therapists working in psychological services, ACT+ was seen as a ‘really useful therapy’ because it enables the person to focus on what is important in their own life, which was regarded as particularly apposite for people living with and beyond cancer.	*Because it [ACT*+*] allows you to…Well, it encourages you to live with it really. To get on with it, to rationalise it, and to make the most of the other stuff…And not to dwell on the bad stuff. [PAT.FG2, R1]*
		*And so, I find it a very freeing kind of model. Again, having the emphasis on values, because its very, very person centred. […] Because actually if somebody's had a really difficult experience or a life transition, it's often the point in life where people rethink priorities and have a bit of a different take on what's important in life. (PSY.Int 11, R2)*
ACT+ Integrated options: work/meaningful occupation and exercise/physical activity	In terms of integrating options to support work/meaningful occupation, patients generally acknowledged that there was benefit in incorporating some structure into one's day in the post‐treatment phase. Similarly, patients were generally positive in incorporating issues of physical activity into therapy but flagged that ‘exercise’ could be perceived as meaning gym work, which could be a turn‐off. They suggested using terminology like ‘physical activity [rather] than exercise’, which patients could identify with (*FG2, R3*). Patients also had some anxieties about doing exercise and physical activities (e.g., how much and which type of exercise is safe to undertake). HCPs spoke about the importance of encouraging patients to exercise but some of them admitted they lacked confidence in dealing with this issue well.	*…because I'm self‐employed, I've not been able to work. I've started just doing little things. Not much, because I've got [no energy]…And that seems to be taking my mind off what was bouncing around [in my head]. (PAT.FG1, R3)*
		*When I was going through chemo, my oncologist was brilliant, and what she did [say] to me was get out and do sweaty walking, because that was her phrase. Sweaty walking. Because that will help you with your symptoms. So immediately you're thinking okay, fine, I'm not going to make it worse. Neuropathy was one of my main things. But I then knew that it was okay to do it. (PAT.FG3, R5)*
		*Because I think we're quite good at sort of encouraging patients to do exercise. But when it comes to actually what that entails, it can be quite difficult. And I think a lot of people, myself included, maybe aren't so confident in prescribing exercise and [knowing] how much is too much. (HCP.Int 2)*

### 3.3 Strand 3—ACT+ Training and Intervention Pre‐Pilot Testing

#### 3.3.1 Qualitative Study With Patients and Therapists Post ACT+ Delivery

##### 3.3.1.1 Therapist Perspective

From the analysis of the therapist interviews, three themes were identified: ‘Delivering ACT+ as part of a research study’, which highlights the specific, bounded context within which the therapy was delivered; ‘Covid time’, which locates the study at a particular timepoint when normal NHS services were disrupted as a global pandemic hit; and ‘ACT+ and therapists' practice’ which gives an indication of the potential for ACT+ to be delivered within existing NHS talking therapies services.

##### 3.3.1.2 Delivering ACT+ as Part of a Research Study

Therapists were mindful of following the therapy protocol so that ACT+ was delivered correctly. For one therapist, this was the cause of some anxiety from the outset:

And obviously it's a trial. So, you want to keep it as faithful to the protocol as possible without adding other extra things that you normally might have done with a person who's not part of the trial.

[TH3]

Others described having similar concerns during therapy delivery, including uncertainty about the degree of flexibility within the protocol, particularly in relation to being responsive to the client in the therapy session:

My main difficulty was around how much flexibility there is to adapt what we're meant to be covering each session to what the client's bringing with them and how much flexibility there is to bring different parts of the whole treatment protocol forward in sessions.

[TH6]

Therapists reported talking through these issues at the ACT+ supervision sessions and getting clarity about how they could use the protocol flexibly, and in terms of doubts about ‘am I doing this right?’, feeling reassured that, as one therapist put it, ‘I was kind of on track’ [TH4]. The ‘pressure of the protocol’ was also mentioned [TH2], but with this implicit reference to delivering ACT+ correctly, there is a temporal dimension, as in this instance the therapist was highlighting the importance of delivering therapy within the specified number of sessions.

The allocation of just one participant as an ACT+ client in the pre‐pilot was seen by therapists as limiting their opportunity to get used to the ACT+ model, and as one pointed out, it also limited the feedback they could offer on any possible modifications to the model:

If I was to have another person I could probably comment more in the future.

[TH1]

One aspect of the study that therapists would have preferred to have been different was the length of time between the ACT+ training and being allocated a client. For some, the ‘big gap after the training’ was seen as adversely affecting their confidence in applying their learning. However, it was acknowledged that it might be difficult to match the timing of the training and client allocation in advance, given the study involved large institutions and was dependent on individuals signing up to receive the intervention. Furthermore, the ‘catchment area’ eligibility criterion of talking therapy services (the intervention delivery sites) was recognised as a factor:

Because there was such a big gap, there were months in fact. So, you forget the training. I mean I did have the handouts and obviously we could use our initiative…So, we could motivate ourselves and be proactive in that. But I think there is too much of a gap. But you can't decide when we're going to have a client that comes to our service that lives in the area, that we're going to start seeing.

[TH2]

Some therapists highlighted a need for in‐house support to better enable their participation in the study, for example, by having a reduced clinical workload and protected time to attend ACT+ supervision sessions. The core issue was the time pressure on individuals, which therapists suggested should be explicitly recognised at organisational sign‐up to the study.

And on top of all the other service demands, it felt like I was putting in quite a lot of extra time of my own, on top of extra time for other parts of the service. And I don't know if that needs to be more upfront at the beginning of the study, that people agree, or the service agrees that it's more than the fifty minutes allocated time. That there's extra work which is expected and there's expected allocated time for supervisions.

[TH6]

##### 3.3.1.3 Covid Time

Participation in the pre‐pilot study coincided with the onset of the Covid‐19 pandemic for some therapists, and for them, the impact of the pandemic on the delivery of ACT+ was seen in a wider context of service disruption.

…we were trying to work out how we could continue our services, making sure everybody had all the equipment that they needed at home, for instance, before they could start seeing people again. So, some of that has been quite a disruption to be able to deliver the intervention as normal.

[TH4]

Flagging up that the pandemic had necessitated a move to remote working, one therapist reflected on their concerns at the time about delivering ACT+ correctly:

…and I was thinking oh okay, I'm ready for face‐to‐face. But then the pandemic hit. How can I make this more effective? And make it work.

[TH5]

Therapists highlighted how having to adjust the mode of therapy delivery affected clients' engagement with ACT+. In one case, it manifested as an abrupt ending as the therapist reported their client opted to pause therapy when face‐to‐face sessions were no longer an option. In contrast, a therapist described how they supported their client through the transition from face‐to‐face to online therapy sessions, working on maintaining the therapeutic relationship through telephone contact while the technology for online ACT+ sessions was set up.

In my intervention with this client, she was happy to carry on because I think I persisted in trying to stay in touch with her during the period, and I think she valued having that contact.…So yeah, I think [the pandemic] did really affect things practically but also how connected the patient was to the intervention and the therapy relationship as well.

[TH4]

##### 3.3.1.4 ACT+ and Therapists' Practice

Aside from the ACT+ training they received through participation in the SURECAN study, therapists acknowledged some familiarity with ACT. For example, some therapists cited previous training in ACT, and one referred to an awareness of ACT and having ‘stolen bits and bobs from ACT in the past’ [TH7]. However, it was not simply ACT‐related learning that was being referenced, as therapists highlighted ways in which their work drew on ACT. One example was the long‐term conditions groups which a therapist ran using an ACT protocol.

So, it's based on the psychological processes, fusion, defusion, using mindfulness as a technique. Goals, value based…setting goals based on values, deciding what's…well, looking at values, what's important, setting goals based on values. So those things…I've used them in therapy, they're good for people with long‐term health conditions.

[TH2]

Therapists also reported using ACT to enhance their CBT work, such as through integration of ‘the principles of ACT into CBT’ [TH1] or by ‘using elements of ACT to boost my CBT with people’ [TH4].

Given that ACT could be used to inform therapists' work, as the examples illustrate, it is perhaps unsurprising that therapists reported a good fit of the ACT+ intervention with their way of practising. In addition, some therapists particularly valued the opportunity afforded by participation in the pre‐pilot to use a therapy that was exclusively ACT‐focused.

But doing pure ACT, it was quite helpful to try and just do some pure ACT without trying to link it to CBT with these patients. And I think being part of the trial gave me permission to do that in [an NHS Talking Therapies] setting, so this was quite nice to be able to, yeah, fully immerse myself in an ACT protocol. And I think fits quite well with the client group I worked with.

[TH4]

This positivity about being involved in the study was echoed by other therapists, who saw it as a ‘fantastic learning experience’ [TH6], particularly in terms of trying a new approach, developing skills and working with a new client group (people with cancer).

…it's been a valuable thing to be involved with…in terms of learning about a new way of doing things, a new client group.

[TH7]

One therapist, a self‐declared fan of ACT, was in favour of more ACT‐focused interventions in the NHS.

But it's quite nice to have a pure…an ACT approach if it makes sense, a specific one where you can dig a bit more deeply into that approach. Because I think some people really benefit from that.

[TH1]

Another therapist who had limited opportunity to deliver ACT+ due to their client withdrawing early on, explained that they had not been thwarted as they had applied their ACT+ learning in a slightly different context.

I think it's a really lovely model. Even though I perhaps haven't fully delivered ACT+ I've been finding that I've been working in that way with my own patients. That's not on the trial. But yeah, I think it could really work well. Especially with long term health conditions.

[TH3]

For this therapist, the fit of ACT+ with their everyday practice was not something merely to have a view on; rather, it was enacted.

##### 3.3.1.5 Patient Perspective

Analysis of patient interviews identified two overarching themes. The first theme, ‘Acceptability and perceived impact of ACT+’, captures participants' views on the therapy, including its appeal, helpfulness and any perceived changes resulting from the sessions. The second theme, ‘Engagement with ACT+’, highlights factors influencing participation, including barriers and facilitators to engagement, as well as suggestions for adapting the intervention to better meet patient needs.

##### 3.3.1.6 Acceptability and Perceived Impact of ACT+

Patient participants were able to understand ACT+ and its principles. They viewed the therapy as a means of fostering acceptance, helping them come to terms with their cancer experience while learning to live with difficult thoughts and emotions.

I understood it (i.e., the therapy) as you're accepting that it's happened, and having the confidence to move forward with it, rather than from it.

[PT2]

Many found the techniques and strategies used in ACT+ both intuitive and beneficial. In particular, the focus on individual values was well received.

I liked that it focused on what's important to you.

[PT2]

Metaphors used in therapy, in particular, helped some participants shift their standpoint:

(the metaphor) really focused me; it made sense. I don't remember it fully, but I definitely think that had changed the way I was thinking at the time. And then this has carried on.

[PT3]

However, not all participants appreciated similes, with one describing a metaphor as ‘simplistic’ [SHEI0064] and difficult to relate to. Similarly, while some appreciated having a handbook as a reference tool, others felt it was unnecessary or overwhelming, noting that ‘a talking basis was better’ [PT2].

Despite varying individual preferences, participants described therapy sessions themselves as beneficial overall, with some reporting lasting changes in their outlook and coping strategies:

I think differently now. They showed me a different way for my problem, and it was good.

[PT6]

It probably gave me some ideas into…If you've got say a negative thought, how you can move that across in your brain and then work on something else.

[PT5]

Others highlighted the value of speaking with a professional as opposed to family members, finding it helpful for processing their thoughts and emotions:

You can speak with that person and it's not your husband, it's not your son. And it's a different person. And he gave me some advice and some ways to calm down and to think about my situation. So for me it was very, very positive.

[PT6]

However, external factors such as the COVID‐19 pandemic were mentioned as barriers to fully assessing the therapy's impact, as lifestyle restrictions influenced participants' experiences also.

##### 3.3.1.7 Engagement With ACT+

Participants' engagement with ACT+ therapy varied. While some reported a smooth therapy experience, others cited logistical and technical challenges that affected participation. The shift to remote delivery due to COVID‐19 introduced difficulties that disrupted scheduling and engagement.

We started face‐to‐face of course, three sessions I think. But then we tried…the COVID problem started and then we started by phone. But I don't have a good connection and I don't listen properly, sometimes the call goes down and then we stopped the sessions.

[PT6]

While some participants valued the convenience of not having to travel, others struggled with establishing privacy at home or found remote sessions less engaging than in‐person therapy. However, video calls were generally preferred over audio‐only sessions, as they helped participants feel more connected.

I suppose it's (i.e. video) better than not at all, and for me it's better than phone. Some people love the anonymity just of phone. I'd prefer a face‐to‐face. The only really good thing about it is it means you don't need to travel.

[PT2]

Additionally, some participants faced personal challenges, including ongoing physical symptoms, medical complications and broader issues that made it difficult to prioritise therapy.

I just said I can't do this […] I'm sure I would've got a lot more out of it […] But I think…because there were a lot of other things going on in the family and I think I just concentrated on that and not myself.

[PT4]

Regardless of such barriers, many participants emphasised that a strong therapeutic relationship was key to engagement in ACT+. Many praised their therapists for being empathetic, patient and attentive.

*She was lovely and there were times where I did get upset, and yeah, she was lovely. [PT3]*

*She was so nice. She had a lot of patience with me. […] She was willing to let me open up and talk about everything. [PT4]*

Therapist skills in adapting to individual needs were also valued, making participants feel understood and respected.

When questioned about potential improvements to ACT+, participants generally expressed satisfaction with the therapy, with several stating that they had no specific suggestions. Sessions were seen as well‐structured, although some highlighted the importance of flexibility in tailoring therapy to individual needs and circumstances.

### 3.4 Intervention Considerations and Refinements

Intervention refinements were informed by insights from all strands of the research, including evidence from the meta‐ethnography, qualitative findings and input from PPI activities. See Supplementary Information File 3 for a detailed timeline of changes and changes in detail.

#### 3.4.1 Acceptability of the ACT+ Intervention

From the thematic analysis of interviews at the end of the pre‐pilot study (Strand 3), it is evident that the response to the ACT+ intervention was positive from both the patients who had the therapy and the therapists who delivered it (Strand 3). Similarly, stakeholders who took part in our first qualitative study (Strand 2) viewed ACT+ as a therapy that could be beneficial for people living with and beyond cancer. Changes and additions were primarily made to intervention resources (i.e., participant handbook and therapist manual) and the therapist training to emphasise that although ACT+ sessions follow a structure, each session should be planned flexibly depending on patient needs. Similarly, we clarified that the participant handbook, which we organised in stand‐alone chapters, is intended to be used flexibly. Furthermore, the language and imagery used in the participant handbook were adapted and reviewed by PPI as a result of feedback from PPI consultations and interviews with patients.

#### 3.4.2 Information for Therapists About Cancer as a Physical Health Condition

From a team review of therapist interview transcripts across qualitative studies, it was identified that therapists may have concerns about discussing physical health and treatment‐related aspects of cancer in therapy sessions. The team decided to offer therapists some education about cancer as a medical condition and address an issue which may be of concern for some of them: how to manage the intersection of ‘being a therapist’ and ‘dealing with cancer’. We included a session on cancer in the ACT+ training and identified resources (produced by Cancer Research UK; https://www.cancerresearchuk.org) to which therapists were signposted both in training and in the therapist manual.

#### 3.4.3 Integrating Physical Activity and Meaningful Occupation

Given therapists had suggested they needed more guidance on integrating physical activity and discussing issues related to work/meaningful occupation, detailed information was provided in the therapist manual and resources were produced to help facilitate conversation around these topics, that is, the ACT+ work conversation adapted from the Work Ability Support Scale V3.6. The training programme was adapted accordingly by specialists in designing such programmes for cancer populations.

#### 3.4.4 Delivery Mode for ACT+

The team agreed to explore whether delivering ACT+ via telephone or online (e.g., via Zoom) would be acceptable to therapists and patients. The pandemic then struck, and online delivery of therapy was necessitated.

#### 3.4.5 Family Involvement

Although we had produced a leaflet for family and friends about ACT+, we explored usual practice in IAPT to ascertain whether involving family and friends in sessions would fit with their usual practice. Generally, therapists were comfortable with this and agreed to have a discussion with patients about possible involvement of significant others when appropriate.

#### 3.4.6 General Feedback

From a review of the therapist's transcripts, several other issues were identified. Being able to access ACT+ supervision, a desire for more role‐play/live examples of the intervention in action, more options for mindful activity, further guidance around developing and establishing values, and using values cards in remote sessions (i.e., conducted by telephone or online) were highlighted. In response to these issues, we clarified supervision arrangements; supervision was provided monthly, but the option of additional sessions was offered if required. We also developed and provided top‐up training periodically for any therapist delivering ACT+ and included additional role‐plays. We discussed values and ways of introducing them in remote sessions, namely via posting cards to the participants, which worked well based on feedback we received in supervision sessions.

#### 3.4.7 Final ACT+ Intervention

We iteratively adapted the intervention called ACT+. We ensured that it was culturally acceptable to different groups in the United Kingdom and suitable for different cancer groups. We settled on 8 sessions to be delivered face‐to‐face or remotely, depending on patient choice and circumstance. Information was added to the written materials on exercise and meaningful activity. Emphasis was put on the importance of these issues being integrated into conversations about value‐based activities to avoid them becoming prescriptive. The patient handbook and therapist manual will be used as a guide during the delivery of ACT+ and will reinforce the theory of ACT, which, in a nutshell, is focused on the centrality of cognitive flexibility, that is, the ability to respond flexibly to life's challenges whilst in pursuit of realistic value‐based goals. We agreed that supervision would be offered to all therapists monthly, but additional sessions offered when requested. The training programme was adapted to cover all the topics important for the delivery of a complex intervention such as ACT+. The content of the intervention is summarised in Tables 2 and 5. The TIDieR (Template for Intervention Description and Replication) checklist was used to aid clarity in understanding the final version of the intervention (see Supplementary Information File 4) [22].

**Table 5:** Content of ACT+ therapist manual.

Overview of the SURECAN trial Following the SURECAN trial protocol Understanding the experience of cancer Introduction to Acceptance and Commitment Therapy (ACT) Overview of strategies used to target psychological processes ACT+: an overview of the process of treatment Starting the process of doing ACT Encouraging physical activity and exercise goals Encouraging and supporting meaningful occupation/paid work Therapist preparation Overview of the ACT handbook Key Materials

## 4 Discussion

We carried out three strands of work to ensure that ACT+ was developed in a way that was both theory‐driven and acceptable to patients and HCPs. Drawing on the principles of ACT, we developed the initial patient‐facing materials—including the participant handbook and therapist manual—and refined them iteratively based on feedback from PPI contributors, qualitative research findings and existing evidence. We applied NPT as a ‘sensitising framework’ throughout the development, refinement and initial piloting of ACT+ to ensure that the intervention accounted for real‐world contexts and to facilitate its implementation if shown to be effective.

Being mindful of the various cancer specialities and pathways in which patients are seen and where the intervention would be accessed, we used qualitative methods to explore the views of patients and HCPs on the proposed intervention (Strand 2). Our findings indicated that the intervention was deemed valuable across HCPs and people with a range of cancer types. Participants highlighted the importance of ACT+ in addressing the recognised reduction in care following the completion of cancer treatment and the lack of psychological support provided in routine cancer follow‐up clinics. Subsequent qualitative interviews with therapists and patients at the end of the ACT+ pre‐pilot study (Strand 3) indicated that the intervention was deemed acceptable by both groups and could be integrated alongside existing clinical practices. Given the increasing focus on long‐term physical health conditions within NHS Talking Therapies, this intervention could be delivered by trained therapists within those services, as well as by counselling and clinical psychologists working in charities and specialist settings, should it prove to be effective. Overall, what emerged from our research was a shared vision of how ACT+ could be delivered to people after the completion of acute cancer treatment, providing structured support at a time when many patients report feeling abandoned by the healthcare system.

ACT+ builds upon existing psychological interventions for cancer survivors while addressing key gaps in post‐treatment support. Although traditional CBT has been shown to moderately improve QoL in cancer survivors [11], it primarily focuses on reducing distress by challenging maladaptive thoughts. In contrast, ACT emphasises psychological flexibility, making it particularly relevant for survivors managing realistic fears such as the fear that the cancer will reoccur, anxieties about the future and long‐term physical challenges. Furthermore, the proposed intervention integrates options to support exercise and meaningful occupation in line with individuals' values, distinguishing it from other psychological therapies. By integrating these elements within a flexible, values‐based framework, ACT+ aims to address the broad life challenges faced by cancer survivors. Importantly, ACT+ was developed to address a significant gap in cancer care pathways, where psychological support is often limited or lacking once hospital‐based treatment is complete. While initiatives such as the NHS Recovery Package offer holistic needs assessments and signpost patients to services, ACT+ provides a structured therapeutic intervention that directly targets survivors' emotional well‐being and QoL.

### 4.1 Methodological Strengths and Weaknesses

A key strength of this study was the meaningful integration of PPI throughout the intervention development process. Two individuals with lived experience of cancer were co‐applicants in the study, providing detailed feedback at every stage. In addition, we sought input from broader PPI groups that reflected the diverse communities in East London and Sheffield, where the research was conducted. Their contributions were essential in shaping the participant materials and ensuring that the intervention was culturally sensitive and relevant across different patient groups. We also drew on NPT as a framework to guide the intervention's development, which allowed us to consider broader contextual and organisational factors that could influence real‐world implementation. This has the potential to reduce the time needed to translate the intervention into routine practice if it proves to be effective.

However, several limitations should be noted. Although we gathered feedback on the proposed intervention from a wide range of stakeholders (Strand 2), certain perspectives were missing. In particular, we did not interview healthcare managers or commissioners, whose insights into service‐level priorities, resource allocation and implementation could have provided valuable guidance. Additionally, while we made efforts to gather feedback from diverse patient groups, the majority of interview participants across our studies were white, which limits the generalisability of our findings. Future research should explore further adaptations of ACT+ to ensure cultural appropriateness and accessibility for underrepresented populations. Another limitation is that fidelity to the intervention was not formally evaluated during the pre‐pilot study. However, this will be assessed as part of the main trial and accompanying process evaluation to ensure that the intervention is delivered as intended.

## 5 Conclusions

ACT+ represents an innovative, theory‐driven intervention that has been developed with input from patients, HCPs and PPI contributors. The intervention has been shown to be acceptable to key stakeholders, with considerations for implementation embedded from the outset. As per the MRC guidelines for developing complex interventions, we considered the context in which ACT+ may be implemented, ensured that theoretical underpinnings informed its design, engaged relevant stakeholders and PPI throughout, and refined the intervention iteratively based on feedback [23]. Finally, we used the TIDieR checklist to describe our intervention for transparency. We are now testing the ACT+ intervention in a randomised controlled trial, the protocol of which has been published elsewhere [24]. This trial will provide further insight into the feasibility, acceptability and effectiveness of ACT+ in supporting cancer survivors.

## Author Contributions

**Elisavet Moschopoulou:** investigation, writing – original draft, methodology, writing – review and editing, formal analysis, data curation, resources. **Sheila Donovan:** investigation, writing – original draft, writing – review and editing, formal analysis, data curation. **Debbie Brewin:** writing – review and editing, resources. **Stephanie Taylor:** conceptualisation, investigation, funding acquisition, writing – review and editing, project administration, supervision. **Damien Ridge:** investigation, conceptualisation, funding acquisition, writing – original draft, methodology, validation, writing – review and editing, formal analysis, supervision. **Liam Bourke:** investigation, conceptualisation, funding acquisition, validation, writing – review and editing. **Imran Khan:** project administration, writing – review and editing. **Moïse Roche:** writing – review and editing. **Trudie Chalder:** conceptualisation, investigation, funding acquisition, writing – original draft, validation, writing – review and editing, formal analysis, supervision, resources.

## Conflicts of Interest

The authors declare no conflicts of interest.

## Supporting information

Supporting information.

## Data Availability

We have not made the data available for the qualitative interviews as they may have personal identifiable information embedded.
