# Supplementary material for: Development of ACT+: A Novel, Person‐Centred Psychological Intervention Based on Acceptance and Commitment Therapy (ACT) to Improve Quality of Life in Patients Living With and Beyond Cancer
Source: Health Expect. 2025 Mar 26;28(2):e70237. doi: 10.1111/hex.70237 (PMC11946915; doi:10.1111/hex.70237)
Supplement: Supplementary file 1 — Supporting information. [file HEX-28-e70237-s001.docx]

Supplementary files

Supplementary file 1: Topic Guides

1. **Topic guide for interviews/focus groups with healthcare professionals (Strand 2)**

| **Topic** | **Question** |
| --- | --- |
| Reaction to SURECAN / first impressions | **What do you think about the SURECAN programme?**  What is your impression of the overall programme?  Does it make sense to you? |
|  | **Can you anticipate any challenges for ACT/the trial? Challenges setting up the trial**  **Can you envisage any concerns that staff may have around ACT+/ the trial?**  **What would be the best way to recruit for the trial? Or to identify eligible patients?** |
| Thoughts on ACT+ (Coming back to the intervention…) | **What are you initial thoughts about ACT?**  **To what extend does the ACT approach make sense to you?**  Personally or professionally  **To what extent is ACT+ compatible with existing practices?**  E.g. how cancer services are organised, thinking about the multidisciplinary approach, at a service delivery level  To what extend does it make sense to the NHS? |
|  | (We have made a distinction between ACT and our model of ACT+.)  **What do you think about the “+” part in ACT+? The inclusion of exercise and work support** |
|  | **How useful or not might ACT+ be to patients?** |
|  | **Say the trial is positive, how would the intervention fit with what you do?**  **How useful will it be to you?** |
|  | **Would it make a difference to you “who” was delivering ACT?** |
|  | **Thinking about ACT as a philosophy, which boils down to values and flexibility, how well do you think it would work for different groups of patients?**  **(cultural groups, ethnicity, sexuality, cancer types, gender, religion etc)** |
|  | **Can you think of any ways to adapt our therapy in order to better suit patients? For example, we are going to include significant others.** |
|  | **Is there anything else you would like to say?** |

1. **Topic guide for interviews with cancer patients (Strand 2)**
2. Elicit **NHS experiences to-date**: Can you tell us something about your NHS experience? What one word describes your experience of the NHS? [We just want to spend a few minutes]
3. Elicit **previous experiences of receiving talking therapies** [or other kinds of interventions/care if no talking therapy experience].
   1. What worked/did not work?
4. In what ways, if at all, has your **ethnicity, religion/spirituality/philosophy of life, OR your gender, class, sexuality, type of cancer etc.** influenced the care you have received?
   1. How so? Stereotyping? Experiences of discrimination? Did you feel you were treated with respect? To what extent were your beliefs respected?
5. **ACT+ presentation** (hand out slide pack)
6. **Initial thoughts** about ACT+.
   1. How much sense does it make?
   2. What is your understanding of what it is about?
7. **How useful** might ACT+ be?
   1. How might it help patients like you?
8. What might be **the challenges for patients using ACT+**? Challenges for carers?
9. Elicit thoughts on **session content** e.g. ‘physical activity’ component, ‘meaningful occupation’ component etc.
10. Do you think ACT+ **could be improved**? In what way?
11. Thinking of your philosophy, spirituality, religion, **how does ACT+ fit**?
    1. To what extent is it compatible or incompatible with your faith?
    2. Do you have any views / how comfortable are you with mindfulness?
12. There will be a **choice**. Would you want the ACT+ sessions on your own, or would you like to bring someone with you, and who, if so? Would it be advantageous for patients to be able to take significant others to ACT+ sessions? If so, what might the benefits be?
13. Is there **anything else** you want to say?
14. **Topic guide for post ACT+ delivery interviews with patients (Strand 3)**
15. The therapist that you met with offers different talking therapies to patients. What is your understanding of what the therapist delivered in the sessions that you had?
16. Could you tell me what it was like for you, participating in the ACT+ therapy sessions?
17. Has ACT+ made a difference to you in any way? Are there any personal changes that you associate with ACT+?
18. Did your engagement with the therapy take a smooth course, or were there any difficulties along the way?
19. If there were difficulties during your therapy, what was the cause of those difficulties?
20. Were there any practical challenges that affected your participation in the sessions or your engagement with the therapy?
21. (*If ACT+ delivered over the telephone*) Your sessions with the therapist were conducted over the telephone. What are your views on this way of doing therapy?
22. What is your understanding of the purpose of ACT+?
23. Have you any thoughts on how the therapy, or the process of delivering it, could be improved?
24. What are your views on the therapist’s interpersonal skills – the way they communicated and made a connection with you?
25. Have you got any comments on the Participant Handbook? *Prompts: Did you find it helpful? Do you have any suggestions about how it could be modified?*
26. (*If any ACT+ sessions included significant others*) You invited a relative/friend to attend your therapy sessions. How did that go? Were there any particular issues arising from their involvement in the sessions?
27. Our aim is for ACT+ to appeal to a wide range of people. Is there any particular kind of person, or patient, who might find it difficult to use this therapy? [*Prompts RE diversity: ethnicity, sexuality, cancer type / illness, gender, religion*]
28. Did the Covid-19 pandemic affect your therapy in any way?
29. Is there anything else you would like to say?
30. **Topic guide for post ACT+ delivery interviews with therapists (Strand 3)**
31. What are your views on delivering ACT+ in the NHS?
32. What were the practical issues involved in the delivery of ACT+ for the pre-pilot?
33. (*If delivered via telephone*) You delivered the ACT+ sessions over the phone. What do you think about this way of delivering the therapy?
34. As a therapist, how did the ACT+ intervention fit with your way of practising?
35. What do you think the patient/your client got out of the therapy?
36. To what extent did the patient/your client share in the ACT+ sense of purpose? *Prompt: Was there any mismatch between patient/client and therapist?*
37. Were there any issues to do with the patient/your client completing the therapy? [*Ascertain how many ACT+ sessions were delivered*]
38. Do you feel you had enough sessions to deliver the therapy? What would be the optimal number of ACT+ sessions, given the constraints of the NHS?
39. In light of your experience, how could the content of ACT+ be modified? *Prompt: Anything that worked well, or didn’t work well?*
40. Again in light of your experience, how could the ACT+ resources (Therapist Manual and Participant Handbook) be modified? *Prompt: Anything that worked well, or didn’t work well?*
41. And how could the ACT+ training be modified, in light of your experience? *Prompt: Anything that worked well, or didn’t work well?*
42. During the therapy, did any issues arise that were to do with perceived differences between therapist and patient/client – either your perception of them as different to you, or vice versa? [*Prompts RE difference: ethnicity, sexuality, illness, gender, religion*]
43. (*If any ACT+ sessions included significant others*) The patient/your client invited a relative/friend to attend their therapy sessions. How did that go? Were there any particular issues arising from their involvement in the sessions?
44. How can aspects of ACT+ that worked well be maximised? *Prompt: Any changes needed?*
45. How confident were you in terms of delivering ACT+?
46. Could you tell me about any anxieties or apprehensions you had as the therapist?
47. You delivered the ACT+ intervention during the Covid-19 pandemic. Do you think that affected anything?
48. How did you find liaising with the study team? For example, in relation to: i) patient referral; ii) submitting study documentation and audio recordings; iii) frequency of communication; iv) supervision. *Prompts: What went well? What was not so good?*
49. Finally, is there anything else that has come to mind from our discussion today?

Supplementary File 2: ACT+ Logic Model


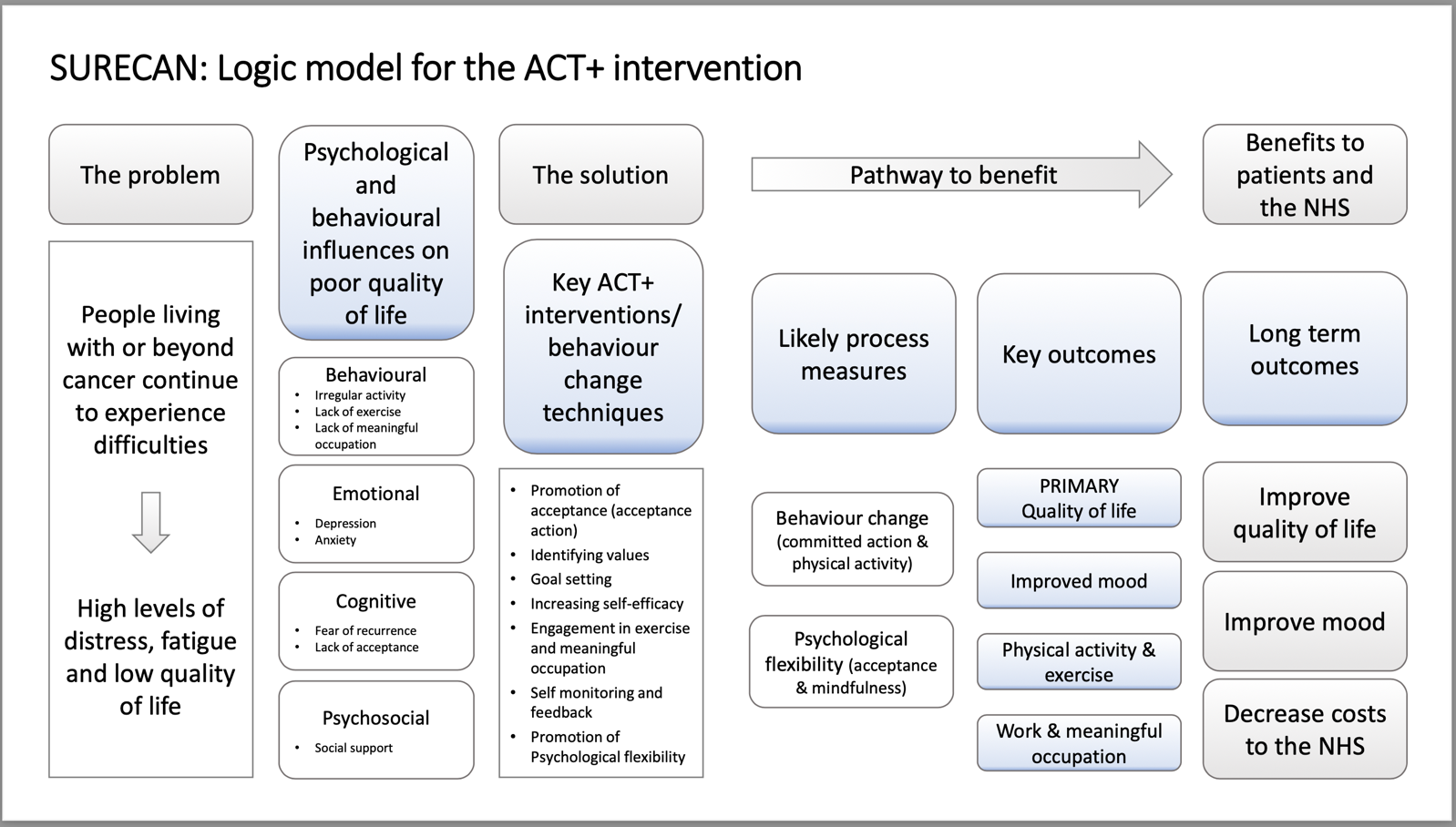


Supplementary file 3: ACT+ Development: Timeline and track changes

Changes made to the ACT+ intervention and associated materials as part of the iterative development process include:

- **Amended intervention manuals and materials as per the feedback we received from patient representatives, therapists and members of the research team:**
  - Included further information on the theory of ACT in the therapist manual
  - Amended the therapist manual to reflect better the flexibility in using the ACT approach but to also provide structure and help therapists adhere to the proposed model
  - Increased the number of therapy sessions to up to 8 as per the feedback of IAPT therapists after training
  - Amended the structure of the participant handbook in order to allow participants and therapists to use it more flexibly (i.e. chapters to choose and work from rather than following a set session by session structure)
  - Amended therapy aids and prompts such as the “What Works Plan”
  - Amended parts of the participant handbook to make it suitable for people from diverse backgrounds (e.g. language and metaphors)
  - Paid attention to the language used in the participant handbook in order to avoid terms such as “mental health” - feedback suggests stigma around certain terms and this could impact recruitment and participation to the study
  - Updated the imagery of the participant handbook in line with patient suggestions – PPI.
  - Included information in the therapist manual on fatigue management as therapists in ACT+ training identified this as an important issue for cancer patients
  - Included in the therapist manual the results of the cultural meta-ethnography aiming to highlight common cultural influences on psychological therapy and implications for ACT+ therapists.
  - Prepared a leaflet for participants’ family members and friends to explain what ACT+ is about and provide options for support should they need it. Evidence from interviews with healthcare professionals suggested that family involvement might be very important.
- **Made changes to the ACT+ training package**
  - Increased duration to 3 days and made sure to leave enough time for reflection and discussion
  - Included multiple speakers who could expertly cover aspects of the ACT+ intervention such as supporting exercise and occupation goals, as well as being aware of cultural influences in psychological therapy and implications for therapists
  - Amended the knowledge and confidence questionnaire in order for the results of this questionnaire to yield more meaningful findings
  - Included more videos, skill demonstrations and role plays
  - Invested more time in explaining the ACT approach
  - Covered common mistakes when using ACT and suggested solutions
  - Included a session at the beginning to address therapists’ experiences of cancer acknowledging that people might have personal experiences of cancer. Interviews with HCPs were supportive of this.
  - Included more information on cancer statistics (e.g. incidence, survival, treatments, side effects) in order to better educate our therapists with regards to understanding a patient’s journey with cancer. Evidence from interviews with HCPs were supportive of this.

Supplementary file 4: Tidier Criteria

| **TIDieR criterion** | **SURECAN description** |
| --- | --- |
| 1. Brief name:   *Provide the name or a phrase that describes the intervention* | Acceptance Commitment Therapy Plus (ACT+) |
| 1. Why:   *Describe any rationale, theory, or goal of the elements essential to the intervention.* | Acceptance and Commitment Therapy (ACT) is an empirically based psychological intervention that aims to increase psychological flexibility. ACT is a ‘third wave’ psychological intervention based on a psychological theory of human language called relational frame theory (RFT). ACT teaches the individual to be in the present moment, to observe thoughts and feelings without trying to change them, and to behave in ways consistent with individual values. The approach should be seen as a way to equip people with helpful coping strategies to enable them to deal with negative thoughts and feelings that may occur in everyday life. The intervention supports and contributes to psychological wellbeing.  Because both work and exercise have been shown to have physical and psychological benefits for people living beyond cancer, we are integrating ACT with physical activity and work/vocational support, where this is relevant to participants’ values (thus, ACT+). |
| 1. What   *(Materials):   Describe any physical or informational materials used in the intervention, including those provided to participants or used in intervention delivery or in training of intervention providers. Provide information on where the materials can be accessed* | Materials for therapists delivering ACT+:   - ACT+ therapist manual - Work sheets: What Works Plan, Weekly Committed Action Sheet, Value cards - Videos and presentation slides demonstrating ACT skills from ACT+ training   Materials for patients receiving ACT+:   - ACT+ participant handbook including intervention work sheets (i.e. What Works Plan, Weekly Committed Action Sheet, Value cards) |
| 1. What   *(procedures):*  *Describe each of the procedures, activities, and/or processes used in the intervention, including any enabling or support activities* | *Training*  3-day training course on ACT+. In addition, trained therapist can receive a half-day refresher training.  *Sessions*  Participants randomised to the intervention arm are referred to the appropriate NHS Talking Therapy service or charity.  Participants receive up to eight, 50-60 minute long, one-to-one sessions with a trained therapist. These are delivered either face-to-face, online or via telephone – according to the participant’s preference.  The content of the sessions is summarised below:  Stage 1: Assessment, engagement and planning of treatment (sessions 1 & 2)   - Assessment and brief history. Introduction to ACT and how it can help.   Stage 2: Active treatment (sessions 3-6)   - Review of the ACT+ model. Exploring values - What is important to me? - Linking values to goals and taking action - Using skills to overcome challenges and ‘Stuck Loops’   Stage 3: Preparation for discharge (sessions 7 & 8)   - Putting it all together - What next   The full content of the intervention, and skills to deliver it are described in detail in the ACT+ therapist manual.  *Support*  All participating therapists receive monthly group supervision with the lead therapist Trudie Chalder |
| 1. Who provided:   *For each category of intervention provider (e.g. psychologist, nursing assistant), describe their expertise, background and any specific training given*. | Therapist training in ACT+ will be delivered by Trudie Chalder, an expert in both ACT and CBT with experience in ACT supervision, as well as in delivering training sessions to NHS Talking Therapies services. The options for exercise have been developed by Liam Bourke, a scientist with expertise in physical exercise for cancer patients. He delivered training for this specifically. The return to work and vocational activity support was developed and taught to participating therapists by Gail Eva, who has expertise in occupational therapy.  Therapists delivering ACT+ will have attended the three-day ACT+ intervention training, practiced the skills and demonstrated competence. They will receive monthly supervision from a senior trained supervisor. |
| 1. How:   *Describe the modes of delivery (e.g. face-to-face or by some other mechanism, such as internet or telephone) of the intervention and whether it was provided individually or in a group.* | Sessions will be delivered via an online video conferencing platform, or via telephone – according to the participants’ preference  The full 3-day ACT+ training will be delivered in person or online. The half-day refresher sessions will be delivered online only |
| 1. Where:   *Describe the type(s) of location(s) where the intervention occurred, including any necessary infrastructure or relevant features.* | In-person therapist training will take place in either at the host university or one of the collaborating universities. Refresher training will be delivered online  ACT+ sessions delivered face-to-face will take place in local health care settings (e.g. participating NHS Talking Therapy clinics, specialist services, or charities offering counselling).  Therapist are given an encrypted digital recorder and encrypted USB stick to record and store sessions. |
| 1. When and how much:   *Describe the number of times the intervention was delivered and over what period of time including the number of sessions, their schedule, and their duration, intensity or dose.* | Participants in the intervention arm will receive up to eight, 50-60 minutes long, weekly or fortnightly, one-to-one sessions with a trained therapist, lasting a period of 14-20 weeks.  The content of the sessions is described in detail in the ACT+ therapist manual. |
| 1. Tailoring:   *If the intervention was planned to be personalised, titrated or adapted, then describe what, why, when, and how.* | Because both work and exercise have been shown to have physical and psychological benefits for people living beyond cancer, we are integrating ACT with options for physical activity and work support provided these are deemed important by the patient according to their values. Therefore, not all participants receive an identical intervention. Furthermore, intrinsic to ACT is working with individuals’ specific values and problem presentations, thoughts and behaviours.  (See ACT+ therapist manual for full details).  All will be individualised as the therapy has a person-centred approach. |
| 1. Modifications:   *If the intervention was modified during the course of the study, describe the changes (what, why, when, and how).* | No modifications have been made |
| 1. How well (planned):   *If intervention adherence or fidelity was assessed, describe how and by whom, and if any strategies were used to maintain or improve fidelity, describe them* | Therapists delivering ACT+ sessions will attend monthly supervision meetings to develop their skills and receive support. Using a manualised approach to the therapy with structured intervention sessions will also help to promote fidelity. Individual advice and support for therapists is also available as required.  Therapists are also offered refresher training to maintain their skills in delivering ACT+  All sessions between therapists and participants will be routinely audio-recorded on an encrypted digital recorder. At the end of the trial, two independent assessors experienced in ACT therapy, will assess fidelity from a random sample of therapist recordings |
| 1. How well (actual):   *If intervention adherence or fidelity was assessed, describe the extent to which the intervention was delivered as planned* | The randomised trial is currently in progress. Adherence and fidelity will be reported in manuscripts published after trials are completed. |
